# Prognostic role of microRNAs in breast cancer: A systematic review

**DOI:** 10.18632/oncotarget.27327

**Published:** 2019-12-24

**Authors:** Eleni Zografos, Flora Zagouri, Despoina Kalapanida, Roubini Zakopoulou, Anastasios Kyriazoglou, Kleoniki Apostolidou, Maria Gazouli, Meletios-Athanasios Dimopoulos

**Affiliations:** ^1^ Department of Basic Medical Sciences, Laboratory of Biology, School of Medicine, National and Kapodistrian University of Athens, Athens, Greece; ^2^ Department of Clinical Therapeutics, Alexandra Hospital, School of Medicine, National and Kapodistrian University of Athens, Athens, Greece

**Keywords:** breast cancer, microRNAs, prognosis, biomarkers

## Abstract

MicroRNAs (miRNAs) have been found to play an important role in breast cancer, functioning either as potential oncogenes or tumor suppressor genes, but their role in the prognosis of patients remains unclear. The aim of the present review study is to highlight recent preclinical and clinical studies performed on both circulating and tissue-specific miRNAs and their potential role as prognostic markers in breast cancer. We systematically searched the PubMed database to explore the prognostic value of miRNAs in breast cancer. After performing the literature search and review, 117 eligible studies were identified. We found that 110 aberrantly expressed miRNAs have been associated with prognosis in breast cancer. In conclusion, the collective data presented in this review indicate that miRNAs could serve as novel prognostic tools in breast cancer, while the clinical application of these findings has yet to be verified.

## INTRODUCTION

Breast carcinoma is the leading cause of cancer death in women worldwide [1]. According to the GLOBOCAN 2018 worldwide estimates of cancer incidence and mortality, in 2018, about 2,088,849 new cases were diagnosed and approximately 626,679 women were predicted to die from the disease [2]. These data support the need to develop more efficient strategies for preventive intervention, evaluation of therapy, and prediction of prognosis [3].

Undoubtedly, TNM staging is of great prognostic value; however, considering all the limitations of the currently available prognostic strategies, it is overall recognized that new affordable more accurate methods indicative of molecular characteristics of tumors are needed to achieve personalized treatment [4]. Still, it remains difficult to achieve these goals, because of the absence of refined (sensitive and specific) biomarkers for disease monitoring and for addressing breast cancer on an individual basis.

MicroRNAs are a small class of endogenous, evolutionarily conserved, single-stranded noncoding RNAs, with a length of approximately 19–24 nucleotides [5]. Interaction between miRNAs and mRNAs, within the 3′untranslated region of the target genes, leads to the degradation or inhibition of mRNA translation [6]. In the past few years, miRNAs have attracted considerable attention in the cancer research field, due to their regulatory actions in multiple levels [7, 8]. Depending on the target gene that they regulate, miRNAs can either serve as “tumor suppressor miRs” by repressing oncogenes or as “onco-miRs” by targeting tumor suppressor genes. However, a number of miRNAs play both tumor suppressor and onco-miR roles depending on the cellular context and tumor type [9].

Particularly in breast cancer, microRNAs (miRNAs or miRs) have been proposed as promising biomarkers because they can be readily detected in tumor biopsies (non-circulating miRNAs) and can also be identified in blood, plasma, serum, and saliva (circulating miRNAs) [10]. Furthermore, circulating miRNAs are bound to lipoproteins such as HDL, are associated with Argonaute 2 (Ago2) protein, or are packaged into exosome-like microparticles, micro-vesicles, and apoptotic bodies [11]. Therefore, they are protected from endogenous RNAase activity, and hence they are reliable.

Several lines of evidence have proven that in breast cancer, the expression levels of miRNAs are altered due to key mechanisms, such as epigenetic control, transcription factors, or the effect of mutated proteins [10]. According to previous publications [12], miRNAs are considered as tumor suppressive or protective when they are down-regulated in cancer compared to their normal counterpart, or else, they are termed oncogenic miRNAs or onco-miRs. In this context, miRNAs are increasingly recognized as promising biomarkers, given the fact that they are easy to isolate, and they maintain their structural stability under different conditions of sample processing and isolation. A prognostic biomarker should indicate a patient’s outcome, for example disease recurrence or disease progression, independent of the treatment regimen that was followed, and they are highly desirable for personalized or precise patient treatment [13].

The aim of the present review is to highlight recent preclinical and clinical studies performed on both circulating and tissue-specific miRNAs and therefore to identify their potential role as prognostic markers in breast cancer. We will particularly focus on the potential role of miRNAs in breast cancer prognosis, and on how miRNAs have the potential to answer actual clinical needs, such as identification of biomarkers for prognosis, in order to achieve the goal of individualized breast cancer treatment.

## RESULTS

**Figure 1:**
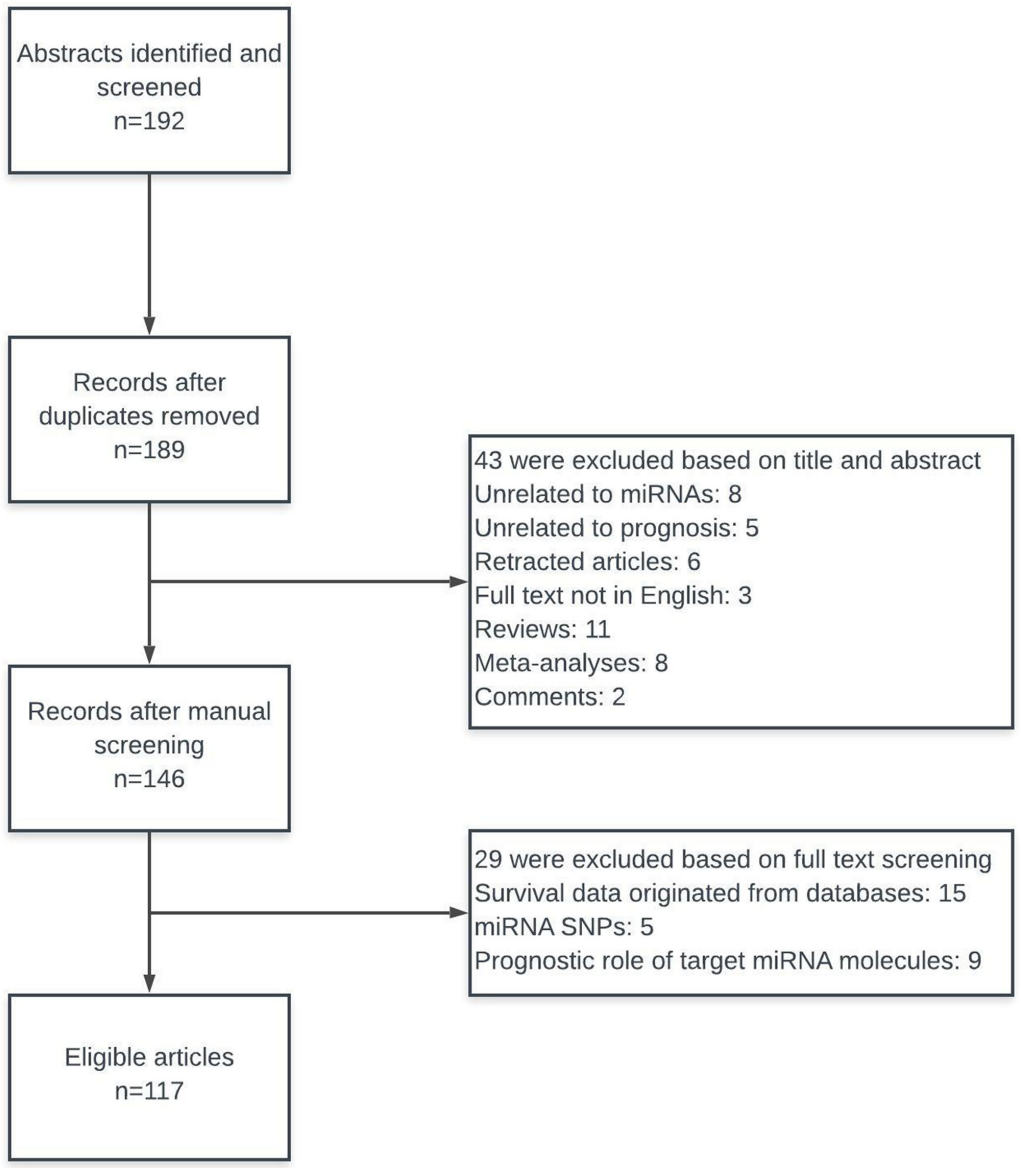
Flow diagram of the study selection process

The search strategy retrieved 192 articles. Of these articles, 42 were irrelevant, 11 were reviews, eight (8) were meta-analyses, six (6) were retracted articles, three (3) were not in English, three (3) were duplicates, two (2) were comments and 117 were eligible. The aforementioned steps concerning the selection of studies are illustrated in detail in Figure 1. Therefore, a total of 117 articles were eligible for this systematic review and the prognostic role of 110 miRNA molecules is described (Table 1). Furthermore, we retrieved five studies, in which authors have identified six distinct microRNA signatures with prognostic value in breast cancer (Table 2).

**Table 1:** List of prognostic microRNAs in breast cancer

Prognostic microRNA	Breast cancer type	Detection method	Prognostic value	Role	Biological sample	References
let-7	BC not classified	qRT-PCR	potential prognostic biomarker as altered levels of miR-let-7 are associated with metastases risk	tumor suppressor	serum	[56]
let-7-3p	TNBC	NGS, qRT- PCR	independent prognostic factor for OS, DFS	onco-miR	FFPE	[57]
let-7b	luminal subtype	qRT-PCR, LNA-ISH, TMAs	independent prognostic factor for OS associated with luminal tumors	tumor suppressor	FFPE	[58]
let-7c/miR- 99a/miR-125b cluster	estrogen- dependent BC cell line	Nanostring, qRT-PCR, luciferase assay	potential prognostic factor for OS in the luminal A subtype	tumor suppressor	cell lines	[59]
miR-1	ER-positive, stage IV BC	PCR, microarray, ISH, IHC	independent worse prognostic factor of DFS and BC-specific survival associated with stage, lymph node metastasis, distant metastasis, histological grade, ER status, PR status and Ki-67	onco-miR	FFPE	[60]
miR-7	BC not classified	qRT-PCR	potential prognostic factor for OS, DFS predictive of an adverse response to tamoxifen therapy	onco-miR	fresh frozen tissue, cell lines	[61]
miR-9	TNBC, BC not classified	qRT-PCR	prognostic factor of DFS and DMFS, OS	onco-miR	FFPE, fresh frozen tissue, cell lines	[62, 63]
miR-10b	BC not classified, TNBC	qRT-PCR	independent prognostic factor for DFS associated with distant metastasis, occurrence in TNBC, associated with genico- obstetric history	onco-miR	FFPE, fresh frozen tissue, cell lines	[17, 40, 41, 64]
miR-15a	TNBC	qRT-PCR	prognostic factor for OS, DFS	tumor suppressor	fresh frozen tissue	[65]
miR-16	triple possitive BC	qRT-PCR, Western blot, luciferase report assay, MTS assay	potentially tumor suppressive effect on cancer progression of ER positive breast cancers, impairment of cell proliferation	tumor suppressor	FFPE	[45]
miR-19a	newly diagnosed IBC stage III, IBC stage IV, non-IBC stage II-IV and HER2+ BC	qRT-PCR	potential prognostic factor for OS, DFS in patients with metastatic HER2(+) IBC.	tumor suppressor	serum, cell lines	[66]
miR-19b	BC not classified	qRT-PCR	prognostic factor for OS associated with distant metastasis and TNM stage	onco-miR	fresh frozen tissue, cell lines	[67]
miR-20b-5p	BC not classified	microRNA arrays	potential prognostic factor for DFS, correlated with the presence of breast tumor interstitial fluid	onco-miR	FFPE, interstitial breast tumor fluids, serum	[68]
miR-21	stage II/III BC, HER2 positive, TNBC	qRT-PCR, microarray, luciferase report assay	independent prognostic factor of OS, DFS, prognostic biomarker for resistance to trastuzumab, to predict lymph node metastases occurrence in TNBC, to predict high grade in non TNBC possible, prognostic factor in daughter of patients, associated with genico- obstetric history	onco-miR	FFPE, serum, fresh frozen tissue, cell lines	[16-27]
miR-22	BC not classified	qRT-PCR, ISH, luciferase report assay	potential prognostic factor for OS, DFS, associated with EMT/metastasis	both	FFPE, cell lines	[69, 70]
miR-24-2*.	BC cell lines	qRT-PCR	associated with tumor suppressive activity through the suppression of cellular survival	tumor suppressor	cell lines, fresh frozen mouse tissue	[71]
mir-24-3p	BC not classified (stage I-III)	Nanostring technology	potential prognostic biomarker of occult metastasis	onco-miR	plasma	[72]
miR-27a	BC not classified	ISH, IHC	independent prognostic factor for OS, DFS	onco-miR	FFPE	[73]
miR-27b-3p	TNBC	qRT-PCR	independent prognostic factor for OS, DMF survival	onco-miR	FFPE	[74]
miR-29a	BC not classified	qRT-PCR, microarray	asocciated with poor response and chemotherapy resistance	onco-miR	FFPE, cell lines	[75]
miR-29b	lobular and ductal subtypes	qRT-PCR	prognostic factor for OS, DFS	tumor suppressor	fresh frozen tissue	[76, 77]
miR-30a	TNBC	NGS, qRT-PCR, microarray, luciferase assay	independent prognostic factor for OS, DFS	tumor suppressor	FFPE, cell lines	[57, 78]
miR-30a-3p	TNBC	qRT-PCR	prognostic factor for OS, RFS	tumor suppressor	FFPE	[57]
miR-30a-5p	TNBC	NGS	prognostic factor for OS, RFS	tumor suppressor	FFPE	[57]
miR-30c-5p	TNBC	qRT-PCR	prognostic factor for RFS	tumor suppressor	FFPE	[57]
miR-30e*	ESR1-/ ERBB2- tumors	microarray, ISH	prognostic factor for DFS	tumor suppressor	fresh frozen tissue	[79]
miR-34a	BC not classified TNBC	qRT-PCR, TMAs	prognostic factor for OS, associated with response and chemotherapy resistance	both	FFPE, plasma, cell lines	[75, 80, 81]
miR-34b	TNBC	qRT-PCR	prognostic factor for OS, DFS	onco-miR	FFPE	[82]
miR-34c	TNBC	qRT-PCR	independent risk factor for OS	tumor suppressor	Plasma	[81]
miR-93-5p	BC not classified	microRNA arrays	potential prognostic factor for DFS, correlated with the presence of breast tumor interstitial fluid	onco-miR	FFPE, interstitial breast tumor fluids, serum	[68]
miR-95-3p	TNBC	qRT-PCR	prognostic factor for OS, RFS in patients treated with anthracycline-based chemotherapy	onco-miR	FFPE	[57]
miR-96	BC cell lines	qRT-PCR	potential prognostic factor for OS associated with EMT and regulation of growth factors involved in G1/S-phase transition	onco-miR	cell lines	[44]
miR-99a	BC not classified	qRT-PCR	potential prognostic factor for OS, independent risk factor for breast cancer	tumor suppressor	serum	[83]
miR-122	BC not classified (stage II-III)	qRT-PCR, NGS	potential prognostic factor for disease relapse, predictor of metastasis	onco-miR	serum	[84]
miR-124	BC not classified	qRT-PCR	prognostic factor for OS associated with advanced TNM stage, lymph node metastasis and poorer pathological differentiation, associated with age at diagnosis (>50 years old)	tumor suppressor	FFPE, fresh frozen tissue	[85, 86]
miR-125a-5p	BC not classified	microarray, qRT-PCR, luciferase assay, ISH, IHC	potential prognostic factor for OS, progression-free survival (PRS)	tumor suppressor	serum, cell lines	[87]
miR-125b	HER2 positive BC, stage II/III	qRT-PCR, ISH	prognostic factor for OS, DFS, associated with aromatase inhibitor esistant breast cancers	onco-miR	FFPE, serum, cell lines	[26, 88, 89]
miR-126-5p	BC not classified	microRNA arrays	potential prognostic factor for DFS	onco-miR	FFPE, interstitial breast tumor fluids, serum	[68]
miR-127	BC not classified	qRT-PCR	prognostic factor of OS	tumor suppressor	fresh frozen tissue, cell lines	[90]
miR-128-3p	TNBC	qRT-PCR	prognostic factor for RFS	tumor suppressor	FFPE	[57]
miR-129-5p	BC not classified	qRT-PCR, luciferase report assay	potential prognostic factor for OS, DFS, associated with EMT	tumor suppressor	FFPE, fresh frozen tissue, cell lines	[91]
miR-133a	BC not classified	qRT-PCR, TMA, ISH, Luciferase assay	potential prognostic factor for DFS associated with migration and invasion	tumor suppressor	FFPE, fresh frozen tissue, cell lines	[92]
miR-140	BC not classified	qRT-PCR, microarray	asocciated with poor response and chemotherapy resistance	onco-miR	FFPE, cell lines	[75]
miR-141	BC not classified	microRNA arrays, qRT- PCR	potential prognostic factor for OS, PFS associated with circulating tumor cells status	onco-miR	plasma	[33, 34]
miR-143	Triple possitive BC	qRT-PCR, Western blot, luciferase report assay, MTS assay	potentially tumor suppressive effect on cancer progression of ER positive breast cancers, impairment of cell proliferation	tumor suppressor	FFPE	[45]
miR-144	BC not classified	microRNA arrays, qRT- PCR	potential prognostic factor for OS, PFS	tumor suppressor	plasma	[34]
miR-145	BC not classified	qRT-PCR	potential prognostic factor for DFS, OS (3-year survival rate)	tumor suppressor	fresh frozen tissue	[93, 94]
miR-146a	BRCA1- deficient TNBC tumors	qRT-PCR	potential prognostic factor for OS	tumor suppressor	FFPE, cell lines	[95]
miR-148a	TNBC	qRT-PCR, microarray	potential prognostic factor for OS associated with metastasis	tumor suppressor	Cell lines, mouse models	[96]
miR-155	TNBC, BC not classified	qRT-PCR, microarray, luciferase report assay	prognostic factor of DMFS, associated with lymph node metastasis	both	FFPE, fresh frozen tissue, cell lines	[62, 97]
miR-182	BC not classified, TNBC	qRT-PCR	potential prognostic factor to predict lymph node metastases occurrence in TNBC, associated with genico-obstetric history, related with hormonal receptors	onco-miR	FFPE, serum	[17, 98]
miR-183/182/96 cluster	BC not classified	qRT-PCR, ISH	potential prognostic factor for OS, DFS	onco-miR	breast tissues not classified, cell lines	[99]
miR-187	BC not classified	TMA, ISH	independent prognostic factor FOR breast cancer– specific survival (BCSS)	onco-miR	FFPE, cell lines	[100]
miR-193b	BC not classified	microRNA arrays, qRT- PCR	potential prognostic factor for OS, PFS	onco-miR	plasma	[34]
miR-195-5p	BC not classified	microRNA arrays	potential prognostic factor for DFS	onco-miR	FFPE, interstitial breast tumor fluids, serum	[68]
miR-199a-5p	TNBC	NGS	prognostic factor for OS	tumor suppressor	FFPE	[57]
miR-199b-5p	BC not classified (-II stage)	qRT-PCR, assays in vitro	potential prognostic factor for OS associated with TNM stage and lymph node metastasis	tumor suppressor	fresh frozen tissue and cell lines	[101]
miR-200a	BC not classified	microRNA arrays, qRT- PCR	potential prognostic factor for OS, PFS, associated with circulating tumor cells status, potential to detect the onset of metastasis	onco-miR	plasma	[33, 34]
miR-200b	BC not classified	qRT-PCR, microRNA arrays, ISH, TMA, luciferase report assay	potential prognostic factor for OS (independent), PFS associated with advanced clinical stage, metastasis, cell proliferation, apoptosis, cell cycle distribution and circulating tumor cells status, potential to detect the onset of metastasis	both	FFPE, plasma, cell lines	[33, 34, 46, 47]
miR-200c	BC not classified	qRT-PCR, microRNA arrays	prognostic factor of OS, DFS, potential to detect the onset of metastasis, associated with circulating tumor cells status	onco-miR	fresh frozen tissue, plasma	[33, 34, 102]
miR-200c/141 cluster	BC not classified, TNBC	qRT-PCR, CAT reporter assay, siRNA transfection, Western blot	poor prognostic factor in TNBC, promoting metastasis	onco-miR	FFPE, cell lines, xenograft animal model	[103]
miR-203	BC not classified, ER positive BC	microRNA arrays, qRT- PCR, Western blot, luciferase report assay, MTS assay	potential prognostic factor for OS, PFS associated with EMT and circulating tumor cells status	both	FFPE, plasma, cell lines	[33, 34, 44, 45]
miR-203-5p	TNBC	NGS	prognostic factor for OS	onco-miR	FFPE	[57]
miR-203a	ductal in situ, invasive ductal and lobular carcinoma	qRT-PCR	potential prognostic marker associated with increased stage in invasive lobular carcinomas	tumor suppressor	FFPE	[104]
miR-204	BC not classified	qRT-PCR	potential prognostic factor for OS, DFS, correlated with chemotherapeutic resistance	tumor suppressor	FFPE	[105]
miR-205	BC not classified	qRT-PCR, LNA-ISH, TMAs, IHC	potential prognostic factor for OS associated with tumours of ductal morphology, for OS and DFS in early breast cancer	tumor suppressor	FFPE	[21, 58]
miR-206	BC not classified	qRT-PCR, luciferase report assay	potential prognostic factor for OS	both	fresh frozen tissue, cell lines	[94, 106, 107,
miR-210	early first primary BC, TNBC	qRT-PCR, microarray	independent prognostic factor for OS, DFS, associated with poor clinical outcome in ER- positive, tamoxifen-treated BC patients, involved in cell proliferation, migration and invasion, Potential to detect the onset of metastasis prior to clinical diagnosis, associated with circulating tumor cells status	onco-miR	FFPE, fresh frozen tissue, plasma, cell lines (Breast cancer and tumor-educated macrophages)	[33-39]
miR-210-3p	BC cell lines	qRT-PCR	potential prognostic factor for OS associated with EMT and regulation of growth factors involved in G1- to S-phase transition	onco-miR	cell lines	[44]
miR-215	BC not classified	microRNA arrays, qRT- PCR	potential prognostic factor for OS, PFS, Potential to detect the onset of metastasis prior to clinical diagnosis	tumor suppressor	plasma	[34]
miR-218	BC not classified	qRT-PCR	prognostic factor for OS associated with lymph node metastases, higher grades,	tumor suppressor	fresh frozen tissue	[108]
miR-221	BC not classified	qRT-PCR	prognostic factor for DFS, OS, RFS	onco-miR	FFPE, fresh frozen tissue, cell lines	[41, 63, 109]
miR-221-3p	TNBC	qRT-PCR	prognostic factor for DFS	tumour suppressor	FFPE, cell lines	[110]
miR-222	BC not classified	qRT-PCR, TMA	potential prognostic factor related to lymph node metastasis, down- regulation of the estrogen receptor, EMT, tumor progression, poor response and chemotherapy resistance	onco-miR	FFPE, fresh frozen tissue, cell lines	[75, 109]
miR-222-3p	BC not classified	qRT-PCR, microarray	independent prognostic factor for DFS postoperatively	onco-miR	serum	[111]
miR-301a	BC not classified, TNBC	qRT-PCR, microarray, ISH	prognostic factor for DFS, OS	onco-miR	FFPE	[112, 113]
miR-320a	BC not classified	chromogenic ISH	potential prognostic factor for OS for invasive breast cancer	tumor suppressor	FFPE	[114]
miR-324-5p	TNBC	NGS	prognostic factor for OS	onco-miR	FFPE	[57]
miR-329	BC not classified	qRT-PCR	independent prognostic factor for OS	tumor- suppressor	serum, fresh frozen tissue, cell lines	[115]
miR-330-3p	BC not classified	qRT-PCR	potential prognostic factor for OS	onco-miR	fresh frozen tissue	[116]
miR-339-5p	BC not classified	qRT-PCR, TMA, ISH	independent prognostic factor for OS, DFS	tumor suppressor	FFPE, cell lines	[117]
miR-361-5p	BC not classified, TNBC	TMAs, ISH	prognostic factor for DFS	tumor suppressor	FFPE	[118]
miR-365	BC not classified	microRNA arrays, qRT- PCR	potential prognostic factor for OS	miR-365, onco-miR	plasma	[34]
miR-370	BC not classified	qRT-PCR, TMA	potential prognostic factor for DFS	onco-miR	FFPE	[119]
miR-374a	BC not classified, IDC stage II	qRT-PCR, TMAs, Luciferase assay, MTT assays, IHC	potential prognostic factor for DFS, contributes to tumorigenicity and progression	onco-miR	FFPE, fresh frozen tissue, cell lines, xenograft mouse models	[120, 121]
miR-375	BC not classified, stage II-III locally advanced and IBC patients	qRT-PCR, microRNA arrays, NGS	potential prognostic factor for OS, PFS associated with circulating tumor cells status, related to hormonal receptors	both	serum, plasma	[33, 34, 84, 98]
miR-409-3p	BC not classified	qRT-PCR	independent prognostic factor for OS associated with advanced TNM stage, lymph node metastasis, and poorer pathological differentiation	tumor suppressor	fresh frozen tissue	[112]
miR-423	BC not classified	qRT-PCR, microarray	asocciated with poor response and chemotherapy resistance	onco-miR	FFPE, cell lines	[75]
miR-429	BC not classified	microRNA arrays, qRT- PCR	potential prognostic factor for OS, PFS	miR-429 onco-miR	plasma	[34]
miR-451	BC cell lines	qRT-PCR	potential factor associated with cell survival and endocrine resistance	tumor suppressor	cell lines	[123]
miR-454	BC not classified (stage I-III)	TMA, ISH	potential prognostic factor for OS (especially in TNBC) and DFS, associated with response to anthracycline	onco-miR	FFPE	[124]
miR-454-3p	BC not classified	microRNA arrays	potential prognostic factor for DFS	onco-miR	FFPE, interstitial breast tumor fluids, serum	[68]
miR-486-5p	BC not classified	microRNA arrays, qRT- PCR	potential prognostic factor for OS, Potential to detect the onset of metastasis prior to clinical diagnosis	tumor suppressor	plasma	[34]
miR-493	TNBC	TMAs, ISH	prognostic factor for DFS	tumour suppressor	FFPE	[125]
miR-494	node- negative BC	ISH	8.5-fold risk of breast cancer death (association trend-not clinical significance)	tumour suppressor	fresh frozen tissue	[126]
miR-497	BC not classified, TNBC	qRT-PCR, luciferase assay	potential prognostic factor for OS	tumor suppressor	fresh frozen tissue, cell lines, orthotopic mouse models	[127, 128]
miR-548c-5p	TNBC	qRT-PCR, ISH	independent prognostic factor for OS, DFS	onco-miR	FFPE	[39]
miR-574	BC not classified	qRT-PCR, microarray	asocciated with poor response and chemotherapy resistance	onco-miR	FFPE, cell lines	[75]
miR-574-3p	BC not classified	qRT-PCR, NGS	potential prognostic factor for OS, DFS	tumor suppressor	FFPE	[129]
miR-588	BC not classified	qRT-PCR	prognostic factor of OS	tumour suppressor	fresh frozen tissue, cell lines	[130]
miR-590-3p	BC cell lines	qRT-PCR, luciferase report assay	associated with breast cancer cells viability, growth and apoptosis	tumor suppressor	cell lines	[131]
miR-597	BC not classified	qRT-PCR	prognostic factor of OS	tumor suppressor	fresh tissue	[132]
miR-601	BC not classified	qRT-PCR	prognostic factor for DFS associated with cell proliferation and metastasis	tumor suppressor	FFPE, cell lines	[133]
miR-638	BC not classified, BRCA1- deficient TNBC tumors	qRT-PCR	independent prognostic factor for OS associated with lymph node metastasis and TNM stage	tumor suppressor	FFPE, fresh frozen, cell lines	[95, 134]
miR-644a	BC cell lines	qRT-PCR, luciferase report assay	associated with tumor progression and distant metastasis-free survival	tumor suppressor	cell lines	[135]
miR-660-5p	BC not classified	qRT-PCR, NGS	potential prognostic factor for OS, DFS	onco-miR	FFPE	[129]
miR-711	BC not classified	qRT-PCR	independent prognostic factor for OS, DFS, associated with breast cancer cells’ proliferation, colony formation, invasion	onco-miR	FFPE, cell lines	[136]
miR-744	BC not classified	qRT-PCR, microarray	associated with poor response and chemotherapy resistance	onco-miR	FFPE, cell lines	[75]
miR-801	BC not classified	microRNA arrays, qRT- PCR	potential prognostic factor for OS, PFS associated with circulating tumor cells status	onco-miR	plasma	[33, 34]
miR-874	BC not classified	qRT-PCR	prognostic factor for OS	tumour suppressor	fresh frozen tissue, cell lines	[137]
miR-940	IDC, TNBC	qRT-PCR	prognostic factor for OS	tumor suppressor	serum	[138]
miR-1179	BC not classified	RT-PCR	independent prognostic factor for OS	tumor suppressor	breast tissue not classified, cell lines	[139]
miR-1247-5p	BC not classified	qRT-PCR	independent prognostic indicator for DFS, OS	tumor suppressor	FFPE, fresh frozen tissue, cell lines	[140, 141]
miR-1260	BC not classified	microRNA arrays, qRT- PCR	potential prognostic factor for OS	onco-miR	plasma	[34]
miR-1274a	BC not classified	microRNA arrays, qRT- PCR	potential prognostic factor for OS, PFS	onco-miR	plasma	[34]
miR-1274b	BC not classified	microRNA arrays	potential prognostic factor for DFS	onco-miR	FFPE, interstitial breast tumor fluids, serum	[68]
miR-1825	BC not classified	microRNA arrays	potential prognostic factor for DFS	onco-miR	FFPE, interstitial breast tumor fluids, serum	[68]
miR-3178	BC not classified	qRT-PCR, microarray	associated with poor response and chemotherapy resistance	onco-miR	FFPE, cell lines	[75]
miR-4653-3p	HR+ BC (stage I~III)	qRT-PCR	potential prognostic biomarker for DFS for patients treated with adjuvant tamoxifen	tumor suppressor	FFPE	[142]
miR-6780b	BC not classified	qRT-PCR, microarray	associated with poor response and chemotherapy resistance	onco-miR	FFPE, cell lines	[75]

Abbreviations: quantitative reverse transcriptase real-time polymerase chain reaction (qRT-PCR), In situ hybridization (ISH), locked nucleic acid probe in situ hybridization (LNA-ISH), Immunohistochemistry (IHC), epithelial-mesenchymal transition (EMT), formalin-fixed paraffin embedded (FFPE), Next Generation Sequencing (NGS), overall survival (OS), relapse free survival (RFS), disease free survival (DFS), progress free survival (PFS), breast cancer (BC), triple negative breast cancer (TNBC), Inflammatory breast cancer (IBC), Invasive Ductal Carcinoma (IDC), estrogen receptor (ER), progesterone receptor (PR), human epidermal growth factor receptor type 2 (HER2).

**Table 2:** List of prognostic microRNA signatures in breast cancer

miRNA signature	Breast cancer type	Detection method	Prognostic value	Role	Biological sample	References
miR-183-5p, miR-194-5p, miR-1285-5p signature	BC not classified	microarrays, qRT-PCR	potential prognostic factor for OS in young breast cancer patients (age <35 years)	miR-183-5p onco-miR miR-194-5p onco-miR miR-1285-5p tumor suppressor	FFPE	[48]
miR-21, miR- 30c, miR-181a, miR-181c, miR-125b, miR-7, miR- 200a, miR- 135b, miR-22 and miR-200c signature	HR positive, HER2 negative	qRT-PCR	potential prognostic factor for DRFS	10-miRNA-based classifier as a prognostic model	FFPE	[49]
miR-155, miR- 493, miR-30e and miR-27a signature	TNBC	qRT-PCR, IHC	potential prognostic factor for OS associated with taxanes resistance	miR-155 tumor suppressor miR-493 tumor suppressor miR-30e onco-miR miR-27a onco-miR	FFPE	[50]
miR-16, 155, 125b, 374a signature	TNBC	qRT-PCR	potential prognostic factor for OS	miR-16 tumor suppressor miR-155 tumor suppressor miR-125b onco-miR miR-374a tumor suppressor	FFPE	[51]
miR-16, 125b, 374a, 374b, 421, 655, 497 signature	TNBC	qRT-PCR	potential prognostic factor for DDFS	miR-16 tumor suppressor miR-125b onco-miR miR-374a tumor suppressor miR-374b tumor suppressor miR-421 onco-miR miR-655 onco-miR miR-497 tumor suppressor	FFPE	[51]
miR-191-5p, miR-214-3p, miR-451a, and miR-489 signature	BC not classified	qRT-PCR, microarray	independent prognostic factor for OS, DFS	miR-191-5p onco-miR miR-214-3p tumor suppressor miR-451a tumor suppressor miR-489 tumor suppressor	FFPE, cell lines	[52]

Abbreviations: breast cancer (BC), quantitative reverse transcriptase real-time polymerase chain reaction (qRT-PCR), formalin-fixed paraffin embedded (FFPE), overall survival (OS), distant disease-free survival (DDFS), distant recurrence free survival (DRFS).

According to our results, presented in Table 1, the majority of publications have not taken into account the distinct breast cancer subtypes during the development of their research protocol, since in 60.8% of studies breast cancer samples were not classified. The remaining 25.8% focused on Triple Negative Breast Cancer (TNBC) samples or involved Luminal A (5.0%), Luminal B (1.7%) and HER2-positive (1.7%) breast cancer samples. Of note, 5.0% of the selected studies accessed the prognostic value of miRNAs through experiments performed on breast cancer cell lines. Different detection methods, as well as different sample types were used for the detection of the prognostic miRNA expression levels (i.e., paraffin-fixed, formalin-fixed, freshly frozen tumors, plasma or serum). Concerning the detection methods, quantitative reverse transcriptase real-time polymerase chain reaction (qRT-PCR) was used in 35,8% of the eligible studies, while in 21,7% of the studies qRT-PCR was performed along with Microarray analysis. Additionally, next generation Sequencing technologies (9,2%), in situ hybridization techniques (9,2%), luciferase report assays (6,7%) or a combination of various techniques (10,8%) were employed.

## DISCUSSION

We conducted a comprehensive systematic literature review to unfold the utility of miRNA biomarkers that can be evaluated for predicting prognosis in breast cancer patients. We have identified 117 studies that investigate the potential correlation between miRNA profile expression in breast cancer tissue and in the circulation and their possible use as prognostic factors. Interestingly, most of the miRNAs found to be associated with prognosis in breast cancer, were assessed in only a single study. Six miRs (miR-10b, miR-200b, miR-21, miR-203, miR-375, and miR-210) were evaluated in at least four studies and the discussion will be mainly focused on these molecules, based on an effort to merely provide some important information on the most commonly researched molecules in accordance with our systematic literature review.

MiR-21 is one of the most extensively studied cancer-related miRNAs and its aberrant expression and deregulation may play a pivotal role in the majority of cancers [14]. miR-21 may serve as a key regulator of oncogenic processes, including tumor growth, migration, and invasion [15], through targeting the pro-apoptotic phosphatase and tensin homolog (PTEN) and promoting tumor cell proliferation [16]. According to our initial search results, we retrieved 12 studies [16-27] and four meta-analyses [28-31] focusing on the prognostic value of miR-21, which collectively provide robust evidence that miR-21 up-regulation is associated with poor outcomes in cancer patients.

Mir-210 has multiple functions in cancer cells and is involved in angiogenesis, cell cycle regulation, DNA damage repair, mitochondrial metabolism, and immune response [32]. According to our search results, including seven studies [33-39], high expression of miR-210 has been significantly associated with poor survival in patients with breast cancer. Notably, single miR-210 assay has been proposed as an independent prognostic factor in this disease.

Concerning miR-10b, it has been presented as a potential biomarker that could play a predictive role in lymph node metastases occurrence across TNBC and in the incidence of high-grade tumors in non-TNBC cases [17]. Elevated expression of miR-10b in breast tumor tissue samples has been associated with adverse outcome, which is further supported from data derived from in vitro studies [40]. Finally, a survival analysis of 230 breast tissue samples has shown that high levels of miR-10b result to a short relapse free survival (RFS) of breast cancer, acting as an independent prognostic factor of RFS [41]. Our results, emphasize the oncogenic role of miR-10b and indicate that its high expression may be correlated with poor survival in breast cancer, while a recent metanalysis further strengthens our findings [30].

MiR-200 family members function as regulators of the epithelial to mesenchymal transition (EMT), which is one of the initial steps in tumor metastasis [42]. Specifically, miR-200b and miR-203 have both been characterized as tumor suppressors in multiple breast tumor types [43]. However, there seems to be an inconsistency in the existing literature, since we retrieved two studies that have found that higher expression of circulating miR-200b and miR-203 are associated with worse outcome [33, 34], further substantiated by a study on breast cancer cell lines [44]. However, other studies on breast cancer tissue samples and cell lines presented inverse results [45-47]. These discrepancies exhibit the diverse regulatory roles of miR-200 family members, depending on the cellular context and type of biological sample (blood VS tissue), and highlight the potential prognostic impact of these EMT regulating miRNA molecules in breast cancer.

Furthermore, our search retrieved five studies that have found six miRNA signatures to be useful for predicting the outcome of breast cancer [48-52]. Coordinated regulation of multiple miRNAs of potential prognostic value, has helped researchers identify panels of prognostic microRNAs for breast cancer. The discovery of microRNA expression signatures shows considerable promise for determining the prognosis of individuals with breast cancer. Similar miRNA signatures have been identified in a variety of other cancers, including acute myeloid leukemia, chronic lymphocytic leukemia, colon cancer, pancreatic cancer, and non-small cell lung cancer [53]. These reports highlight that this class of RNA molecules is showing substantial potential to be used as prognostic biomarkers for cancer.

Among the limitations of this effort, it should be stressed that this process was essentially driven by the search algorithm, which focused mainly on titles of the published literature, in an effort to provide more relevant results. Furthermore, clear heterogeneity was observed in our results, due to differences in patient characteristics (ethnicity, age, tumor stage, grade and subtype) and the use of different isolation and detection methods, cut-off values for miRNA expression levels, sample preparation methods and sample types (i.e., paraffin-fixed, formalin-fixed, freshly frozen tumors, plasma or serum).

Based on the results of this systematic review, we consider that miRNA detection may address the need for independent, easily accessible, prognostic molecular markers for breast cancer management in clinical practice, by assessing the impact of aberrant miRNA expression on patients’ survival. Our work sums up all the available data on prognostic miRNAs and can also act as a valuable reference point for future studies. Furthermore, while prognostic studies can assist in answering important questions concerning specific patient outcomes, their vigorous and careful design is a necessary condition for ensuring the reliability of results [54]. It should be stressed out that the thorough validation of prognostic factors is a necessary and unavoidable process in order to maximize certainty in predicting future breast cancer patients’ outcomes. Therefore, extensive validation studies focusing on particular miRNAs or miRNA signatures should be performed to relate baseline clinical and experimental variables to outcome. Eventually, all the reviewed molecular studies may help in bringing prognostic miRNAs closer to the clinical practice.

## MATERIALS AND METHODS

### Methods of search strategy and study eligibility

This systematic review was conducted in accordance with the PRISMA guidelines [55] and in line with the a priori protocol agreed on and signed by EZ and FZ. Eligible studies were sought in PubMed without any restriction of publication language; the end-of-search date was January 28, 2019. The following search algorithm was used: breast[ti] AND (carcinoma OR carcinomas OR cancer OR cancers OR neoplasm OR neoplasms) AND (microRNA[ti] OR miR[ti] OR miRNA[ti] OR microRNAs[ti] OR miRs[ti] OR miRNAs[ti]) AND (prognosis[ti] OR prognostic[ti] OR survival[ti] OR outcome[ti] OR mortality[ti]). Eligible articles included studies examining the prognostic role of microRNAs in breast cancer. Only prospective and retrospective studies as well as case reports were considered eligible. In instances where multiple (overlapping) publications stemming from the same study were identified, the larger size study and the one with longer follow-up were included, unless the reported outcomes were mutually exclusive. Authors working independently and blindly to each other in pairs (E.Z., F.Z.) performed the selection of eligible studies; in case of disagreement, consensus with the whole team was reached.

### Data extraction

The extraction of data comprised general information, including the name of the miRNA molecule, the breast cancer type in which its expression was determined, method of detection, the sample type that was used, its prognostic value in breast cancer, its function in cancer (onco-miR or tumor suppressor-miR) and the author-year of publication. Data were independently extracted and analyzed by a pair of reviewers (E.Z. and F.Z.), with one reviewer being blinded to the other; if needed, the final decision was reached by a team consensus.

Eligible literature met the following criteria: (1) measured miR expression levels in tumor or blood samples or human cell lines and (2) only articles in English. Publications were excluded if they had one or more of the following criteria: (1) studies referring to the prognostic role of single nucleotide polymorphisms (SNPs) in miRNA genes affecting their function; (2) studies that refer to the prognostic role of target miRNA molecules (molecules regulated by miRs); (3) studies based solely on a bioinformatics approach or a computational algorithm, with survival data originated from databases without subsequent biological validation and (4) review papers, meta-analyses, comments, letters or duplicate publications.
